# Supplementary figures and images for: Assessment of mouse VEGF neutralization by ranibizumab and aflibercept
Source: PLoS One. 2022 Dec 21;17(12):e0278951. doi: 10.1371/journal.pone.0278951 (PMC9770341; doi:10.1371/journal.pone.0278951)

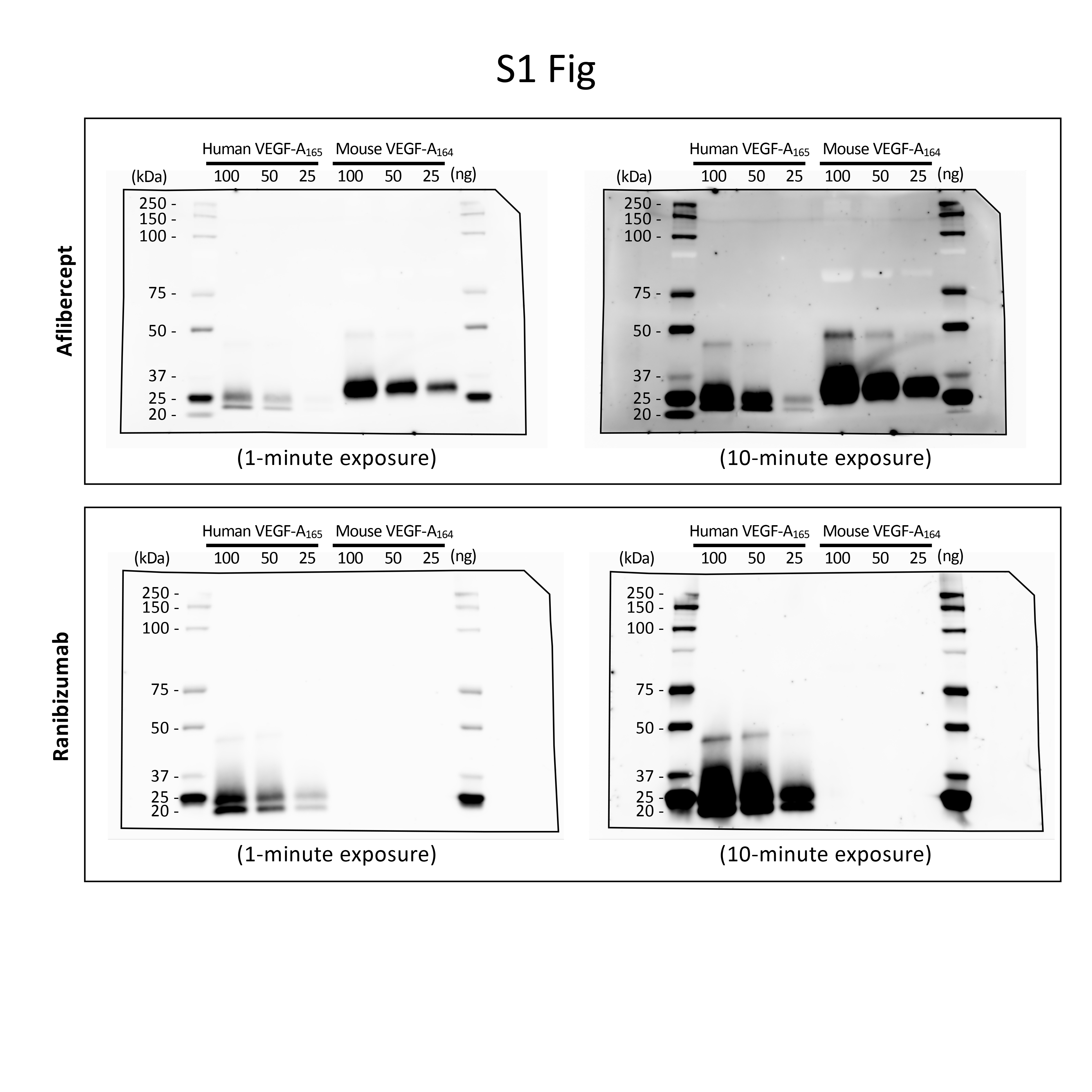

Supplement: S1 Fig — Immunoreactive bands of mouse VEGF-A164 were observed in the aflibercept-probed blot, but not in the ranibizumab-probed blot. VEGF, vascular endothelial growth factor. (TIF) [file pone.0278951.s001.tif]

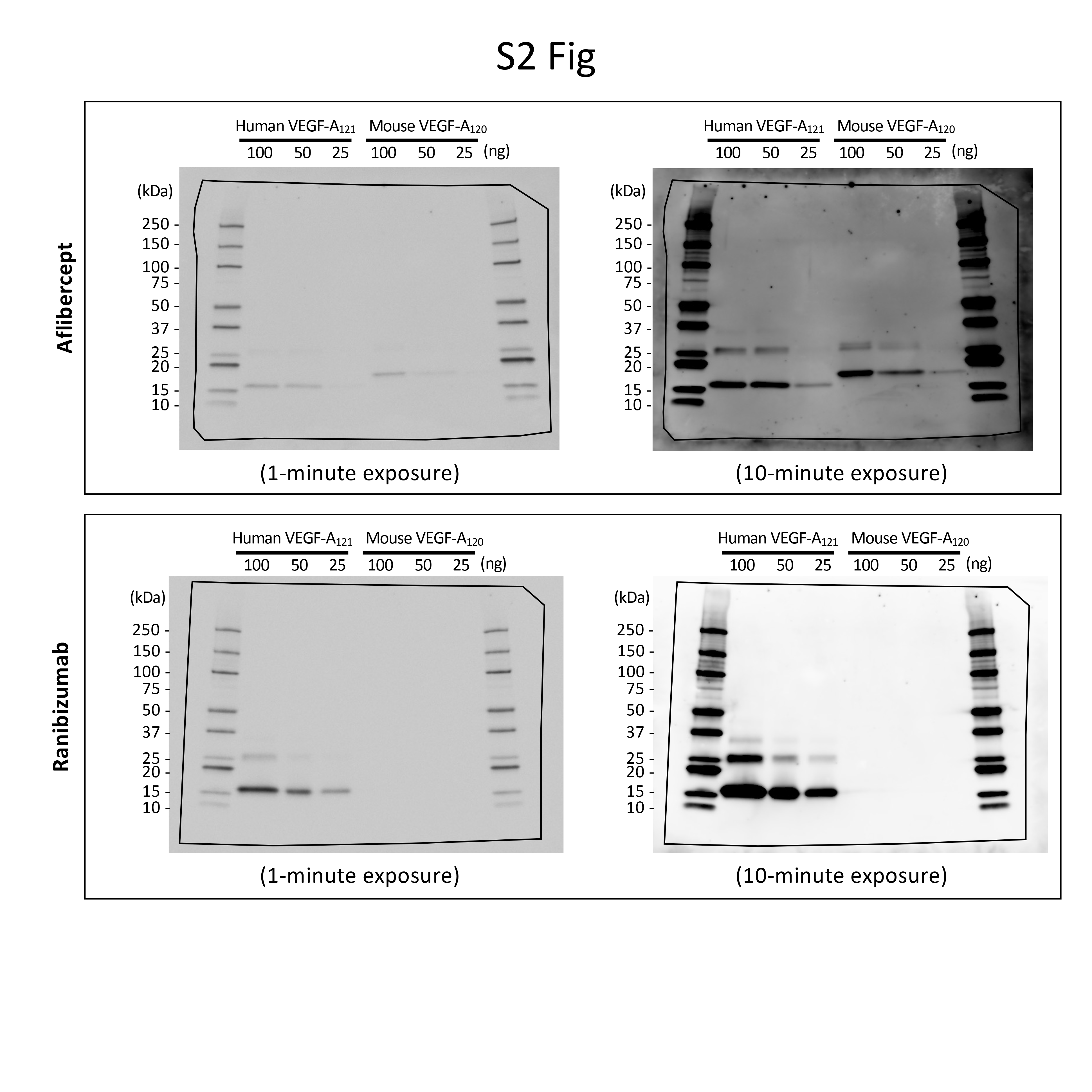

Supplement: S2 Fig — Immunoreactive bands of mouse VEGF-A120 were observed in the aflibercept-probed blot, but not in the ranibizumab-probed blot. VEGF, vascular endothelial growth factor. (TIF) [file pone.0278951.s002.tif]
