# Supplementary material for: Assessment of mouse VEGF neutralization by ranibizumab and aflibercept
Source: PLoS One. 2022 Dec 21;17(12):e0278951. doi: 10.1371/journal.pone.0278951 (PMC9770341; doi:10.1371/journal.pone.0278951)
Supplement: S1 Raw images — (PDF) [file pone.0278951.s003.pdf]

Row images of Fig 3

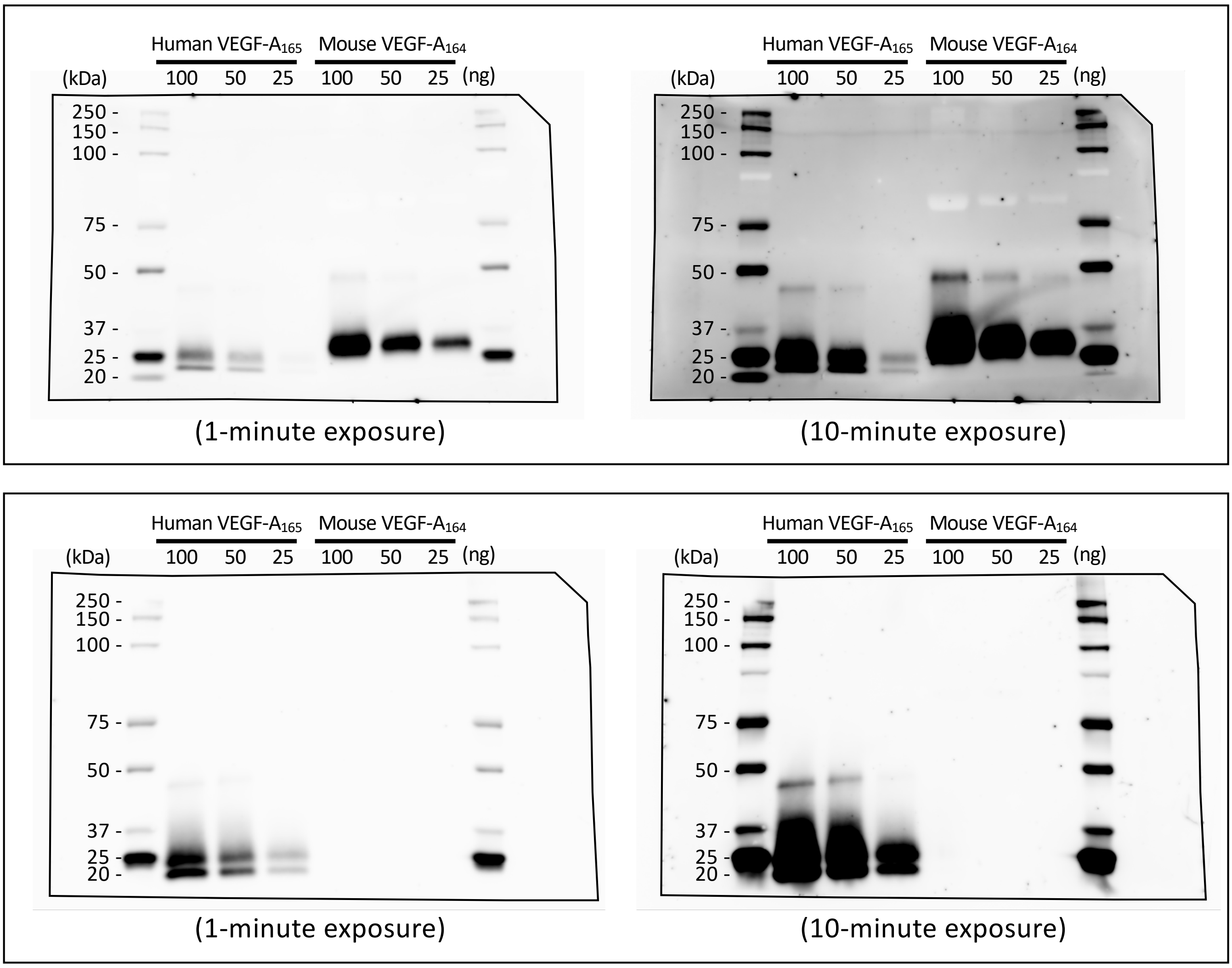

Full-length western blot images of recombinant human VEGF-A_{165} and mouse VEGF-A_{164} detected with aflibercept or ranibizumab as the primary antibody. VEGF, vascular endothelial growth factor.

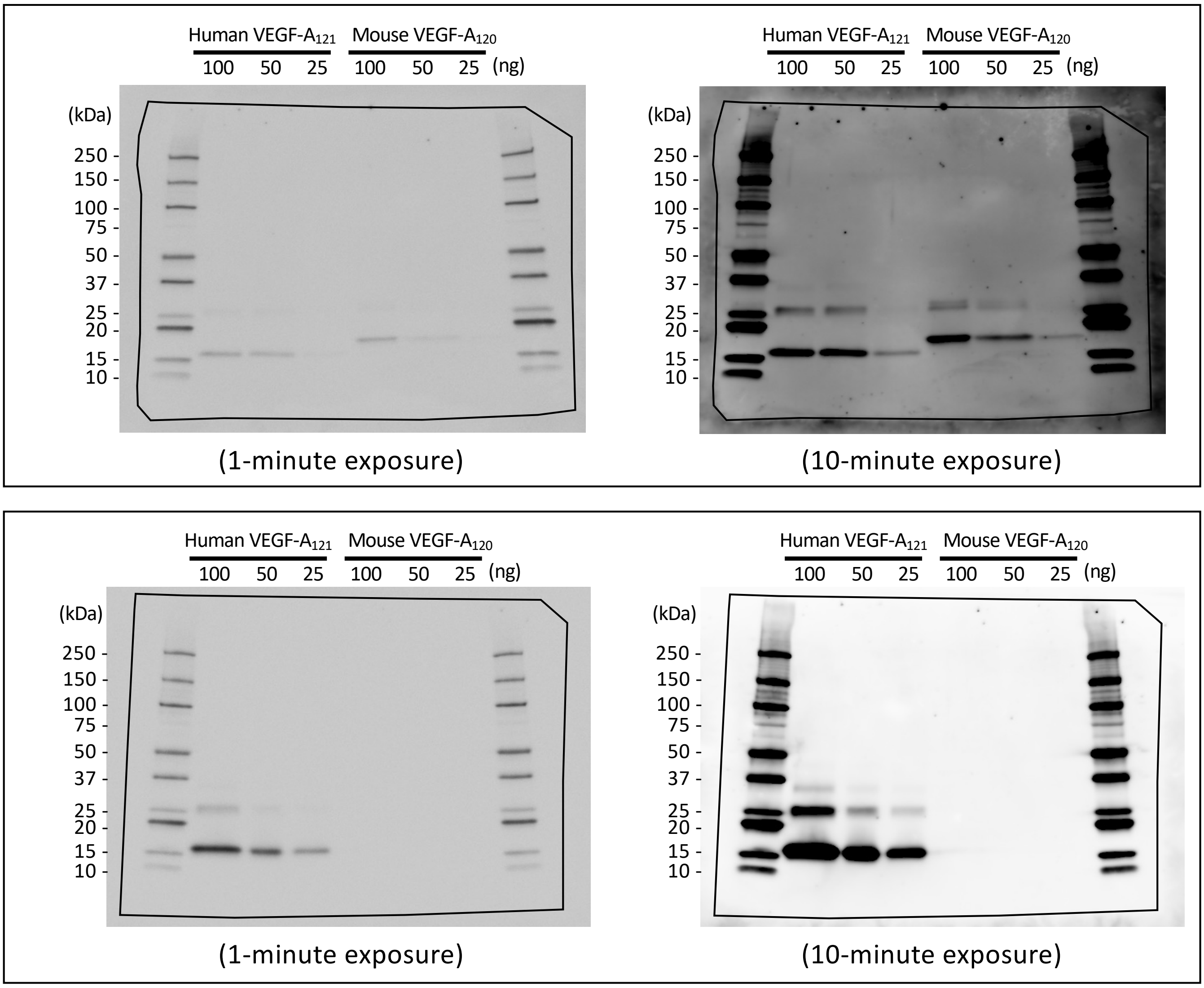

Full-length western blot images of recombinant human VEGF-A_{121} and mouse VEGF-A_{120} detected with aflibercept or ranibizumab as the primary antibody. VEGF, vascular endothelial growth factor.
