# Supplementary material for: Assessment of mouse VEGF neutralization by ranibizumab and aflibercept
Source: PLoS One. 2022 Dec 21;17(12):e0278951. doi: 10.1371/journal.pone.0278951 (PMC9770341; doi:10.1371/journal.pone.0278951)
Supplement: Attachment — Submitted filename: Response to Reviewers.docx [file pone.0278951.s004.docx]

**Response to Reviewer #1:**

- **Authors state that the image-based quantification on this study was made manually with Image J. Please clarify if the investigator working on the images was blinded, since the manual processing could be biased. If any plugin or macro was used, please describe it on “Materials and Methods”. If the investigator was not blinded, this should be stated.**

**Response:** Thank you for your comment. The quantification was performed by a masked investigator. We revised the "Materials and Methods" section as follows.

Page 11, line 153 (Result)

The ratio of the avascular area to the whole retinal area (% avascular area), the ratio of the neovascular tufts area to the whole retinal area (% NVT area), the ratio of the Ter119+ area to the whole retinal area (% Ter119+ area), the distance from the optic disc to the vascular front (radial growth), and total pupillary vessel length were manually measured using ImageJ software by an investigator masked to treatment assignment.

- **Please clarify if PBS was injected on the fellow eye of anti-VEGF injected eyes. The control should be the fellow eye. If that was the case, please separate the PBS column on Figure 1-C, 1-D, 1-E into PBS-injected eyes from Aflibercept treated mice and Ranibizumab treated mice.**

**Response:** Thank you very much for pointing out this important point. We previously reported that intravitreally injected aflibercept migrates hematogenously to the fellow eye, where it exerts its anti-VEGF effect. In this study, therefore, we did not use the fellow eyes of the anti-VEGF injected eyes as control group. To minimize the effects of individual differences, littermates were divided into PBS, aflibercept, and ranibizumab groups. We added the following sentences to "Materials and Methods" section.

Page 8, line 103 (Result)

To minimize the effects of individual differences, littermates were divided into the above three groups.

Page 8, line 111 (Result)

To minimize the effects of individual differences, littermates were divided into the above four groups.

**Response to Reviewer #2:**

- **Methods: Details regarding how many mice were used in total and for each experiment should be provided. In addition, the authors should provide references for the protocols they used for each scientific method and experiment they conducted. (e.g. 87-90, 105-109: The protocols used are missing.)**
- **Lines 105-109, 202-205: The authors should also add references to support the selection of this procedure, otherwise provide a more detailed explanation.**

**Response:** Thank you for pointing out these omissions. The numbers of mice used in total and for each experiment were added to our manuscript. In addition, references to the experimental methods used are indicated, and a detailed description is added as follows.

Page 7, line 86 (Materials and Methods)

Euthanasia for tissue sample collection was achieved by performing cardiac puncture exsanguination under isoflurane anesthesia. The right atrium was then pierced and 5 mL of phosphate-buffered saline solution (PBS) was perfused through the left ventricle. Right after, 5 mL of 4% paraformaldehyde (PFA) at 4 °C was perfused in the same manner to start fixation of the tissues [28]. A total of 46 mice were used for this study.

Page 16, line 226 (Result)

Based on a previous report showing that intraperitoneal administration of 10 mg/kg of aflibercept in developing mice causes severe developmental defects [26], we injected 10 mg/kg of aflibercept intraperitoneally in the aflibercept group. Because 0.5 mg of ranibizumab is usually administered for every 2 mg of aflibercept administered in human clinical practice, the low-dose ranibizumab group received 2.5 mg/kg of ranibizumab, corresponding to one-quarter of the dose of aflibercept. The high-dose ranibizumab group received 10 mg/kg of ranibizumab, corresponding to the full dose of aflibercept.

- **Lines 100-103: The previously described finding that anti-VEGF effects of aflibercept were stronger on P14 could not stand for ranibizumab, too, since it has not been investigated earlier. This fact could affect the findings of the present study and should also be included as a limitation in the discussion section (lines 269-270).**

**Response:** Thank you very much for your important point. We added following sentences in the “Discussion” section.

Page 22, line 333 (Discussion)

A limitation of this study is that we only analyze the OIR retina at 2 days after intravitreal injection of ranibizumab. We used this method because in our previous study [28], we analyzed OIR retinas 2 days and 4 days after intravitreal aflibercept injections and found that the anti-VEGF effects of aflibercept were stronger on P14 than on P16. However, it is unclear whether ranibizumab and aflibercept have the same pharmacokinetics in the mouse eye, and we cannot rule out the possibility that a slight anti-VEGF effect of ranibizumab could have been detected if the analysis had been performed at another time point after ranibizumab injection.

- **Lines 121, 157: Furthermore, they had better clarify which anti-collagen antibody, i.e. coll V or coll IV, they used.**

**Response:** Thank you for your comment. We detected type IV collagen using an anti-collagen IV antibody. To avoid misunderstanding, we changed the abbreviation of type IV collagen from colIV to col4.

- **The authors should include captions for the providing figures and details regarding what each part of the figure, e.g. 1A, 1B, 1C, depicts.**

**Response:** Thank you for your comment. We had listed figure captions at the end of the manuscript under the title "Legends". To meet PLOS ONE's style requirements, we relocated each figure caption to the position directly after the paragraph in which it was first cited. In addition, annotations were added to make it easier to understand what Fig 1B and 1F represent.
